# Prehistoric Plant Exploitation and Domestication: An Inspiration for the Science of De Novo Domestication in Present Times

**DOI:** 10.3390/plants12122310

**Published:** 2023-06-14

**Authors:** Kim Henrik Hebelstrup, Aristotelis Azariadis, Adam Cordes, Peter Steen Henriksen, Henrik Brinch-Pedersen

**Affiliations:** 1 Department of Agroecology, Section for Crop Genetics and Biotechnology, Aarhus University, Flakkebjerg, 4200 Slagelse, Denmark; aristoteles@agro.au.dk (A.A.); adamcordes@agro.au.dk (A.C.); hbp@agro.au.dk (H.B.-P.); 2 Department of Environmental Archaeology and Materials Science, National Museum of Denmark, I.C. Modewegsvej 9, 2800 Brede, Denmark; peter.steen.henriksen@natmus.dk

**Keywords:** fundamentals of agriculture, archaeobotany, breeding, plant-based foods, green transition

## Abstract

De novo domestication is a novel trend in plant genetics, where traits of wild or semi-wild species are changed by the use of modern precision breeding techniques so that they conform to modern cultivation. Out of more than 300,000 wild plant species, only a few were fully domesticated by humans in prehistory. Moreover, out of these few domesticated species, less than 10 species dominate world agricultural production by more than 80% today. Much of this limited diversity of crop exploitation by modern humans was defined early in prehistory at the emergence of sedentary agro-pastoral cultures that limited the number of crops evolving a favorable domestication syndrome. However, modern plant genetics have revealed the roadmaps of genetic changes that led to these domestication traits. Based on such observations, plant scientists are now taking steps towards using modern breeding technologies to explore the potential of de novo domestication of plant species that were neglected in the past. We suggest here that in this process of de novo domestication, the study of Late Paleolithic/Late Archaic and Early Neolithic/Early Formative exploration of wild plants and identification of neglected species can help identify the barriers towards domestication. Modern breeding technologies may then assist us to break these barriers in order to perform de novo domestication to increase the crop species diversity of modern agriculture.

## 1. The Distinction between Wild and Domesticated Plant Species

The shift from hunting and gathering to a sedentary lifestyle with a subsistence based primarily on agriculture was a major event in the history of human ecology, whereby human nutrition became dependent on a few crop species. The cultivation of plants and herding/breeding of animals led to the emergence of domesticated species, adapted to a sedentary human ecology. In Old World archaeology, such events are often referred to as the emergence of Neolithic cultures, where cereals are seen as central to the associated economic transitions [1]. These shifts towards agrarian societies were accompanied by several cultural and material transitions. Neolithic cultures are characterized by sedentary settlements with livestock and plant cultivation. This paved the way for significant changes in material culture, foodways, technology and social organization, such as the invention of ceramics, dairy and cereal products (such as cheese [2,3], bread [4] and beer [5]), the building of megalithic constructions and the emergence of social structures that allowed the rise and development of increasing social inequalities. As such, Neolithic cultures were more complex than those of the prior Paleo-, Epipaleo- or Mesolithic cultures where foraging was the primary basis of the economy. In New World archaeology, a similar transition is called the change from the Archaic to Formative periods.

**Figure 1:**
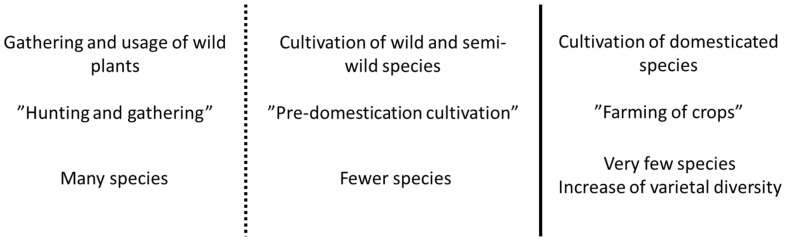
Three-step model for progression of exploitation of plants from gathering to pre-domestication cultivation to actual cultivation of domesticated crops at geographic centers of agricultural origins.

Prehistorical transitions to farming occurred independently in different centers around the globe, where different plant and animal species were domesticated on different continents. However, after the emergence of farming cultures, the access to cultivated species through their geographic origin and particularly through the potential to travel, played a tremendous role in shaping the course of human history [6]. There are several examples where the introduction of new cultivated species into a culture eventually caused significant economic changes. An example of such a historical event is the introduction of the cultivated potato into Europe. Nunn and Qian [7] conducted a systematic analysis to estimate the impact on urbanization and population growth of the introduction of potato into the Old World. Their model suggested that the post-Colombian introduction of the potato into Europe accounted for at least one-quarter of the human population’s growth in the years between A.D. 1700 and A.D. 1900. Plant exploitation in prehistoric times are documented in archaeobotanical records. In general, staple crops in Europe have been introduced mostly from other regions of the world, and the actual emergence of agriculture in Europe is closely associated with the introduction of *Triticeae* cereals from the Near East via a route through Anatolia, throughout the Mediterranean and Aegean Sea and along rivers in the Balkans and into central Europe [8]. In Europe as well as in the Near East, the earliest urban settlements and complex societies with inequality and specialized social classes are linked to the emergence of agricultural economies that thrived upon the production of some of the major crops known today, such as wheat and barley [9]. However, the pattern of transition from a hunter–gatherer ecology based on multiple wild species to a sedentary ecology based mainly on a few domesticated staple crops seems universal. Similar transitions appear to have transpired in other parts of the world, where major crops such as rice and maize play a significant role [10]. However, the oldest agricultural cultures seem to have originated in the Levant, where the origin of cultivation of small-grain cereals around 10,000 years ago was followed first by Neolithic and subsequently by Bronze Age cultures, from where technology, writing, philosophy and trade spread and developed across the Mediterranean area to herald the emergence of literate cultures [6]. In southern Scandinavia, the transition from Mesolithic coastal cultures to Neolithic farmers that occurred around 6000 years before present is specifically associated with the migration of farmers from central Europe [11], who, along with animal husbandry, brought with them the few agricultural founder crops—barley and wheat species—that originated first through domestication in the Near East, but remained the basis of agriculture and economy in Europe up to modern times. Given the impact on economy and urbanization of such access to crop technology and cultivation, it is remarkable that humankind has continued to thrive only upon the limited number of a few existing crops established in prehistory. Archaeobotanical analysis suggests that many species which were partly or fully cultivated in the past were eventually neglected or lost, so that agriculture has become dominated by a few species. The archaeobotanical records also show that the domestication of wild species in western Asia was a process which evolved over a timespan of millennia [10]. Similarly, there are only a few reports on the domestication of new wild species in historic times, of which fewer have shown to be economically competitive with existing major crop species established in prehistory. We know that the cultivation of crops is dependent upon the presence of domestication traits. These traits vary between species. For cereals and some other seed crops, threshability and the loss of seed shattering is pivotal for efficient cultivation [12]. However, traits in the form of physiological adaptations such as seed or tuber dormancy, climatic and geographic adaptations are also important for farming. Fully established crops are often characterized by having passed a point-of-no-return in their evolutionary development. This means that key traits have changed to an extent where they are no longer able to thrive in the wild, and, therefore, survive only because of human cultivation. In short, the key to the success of a crop to fully establish itself in human cultivation is its ability to evolve traits that lead to positive selection under human cultivation. The transition to a species trait that occurs when it shifts from living under natural selection in the wild to living under selection by human cultivation is known as the domestication syndrome. We must assume that the lack of domestication traits represents a barrier for wild plants to be established as crops. In recent years, the cloning of plant domestication genes has shown that several domestication traits are the result of spontaneous mutations that occurred in populations of plants under domestication. To explain the emergence of domestication traits in wild populations, it has been suggested that cultivation before actual domestication (so-called pre-domestication cultivation) plays a significant role. In a model suggested by Weiss et al. [13], human domestication of plants is divided into a gradual shift between three stages. In the first stage, annual plants from wild stands are gathered and utilized as a part of the economy. In the second stage, wild plant genotypes are systematically sown in fields, and in the third stage, fully domesticated plants are cultivated. In the Levant, where several crop species such as small-grain cereals and legumes were domesticated, this model is supported by archaeological finds that suggest a significantly prolonged period using wild cereals before the emergence of Neolithic cultures that had a significant reliance on farming economies with the cultivation of domesticated plant species (Figure 1). During this prolonged period and into the early Neolithic, archaeological grain records show a slow progression in the emergence of visible domestication traits, such as the loss of seed shattering through the emergence of non-brittle rachis and higher yield through enlarged grains [14]. Genetic studies show how, even in prehistory, cultivation favored domestication alleles and increased varietal diversification within a crop species [15]. However, the transition towards modern intensive agriculture has increased the risk of varietal loss of the high agro-diversity known in traditional agriculture.

Archaeological remains of wild cereal use in the Levant before the onset of Neolithic farming cultures can be documented to more than 10,000 years before actual agricultural practices. There are numerous examples of this, and in all cases, wild cereals have been interpreted to have been collected and used for processing and consumption. An important archaeological find that demonstrates activities of bread baking with several species of large-seeded grasses dated to 14,400 years before present was found at the archaeological site Shubayqa 1 in northeastern Jordan [4]. Wild cereal processing on grinding stones also precedes cereal cultivation by many thousands of years. The oldest known evidence dates to almost 23,000 years before present (BP) and has been excavated from the archaeological site Ohalo II in Israel. In one of the huts excavated at the site, a flat basalt stone was found. Starch grain analysis indicates that the stone had been used for grinding wild cereals, primarily wild barley [16]. Dental microwear analysis was carried out on two human teeth from Ohalo II. While one mandible originated from a skeleton, the other mandible represents an isolated find. The observed high frequency of long narrow scratches with a few small pits suggested a tough abrasive diet consisting of stone-ground plant foods. The results further suggested that aquatic foods may also have constituted part of the diet [17].

At the same location, an alignment of burned stones was interpreted to be a hearth-like structure used as a simple oven [18]. Rock-cut mortars are present at numerous sites in the southern Levant that date to the time before agricultural practices, 15000–11700 BP. Experimental studies have shown that these were likely used to produce flour for the production of groat meals, porridge and unleavened bread [19]. This indicates that bread baking may have been practiced more than 20 millennia ago.

**Figure 2:**
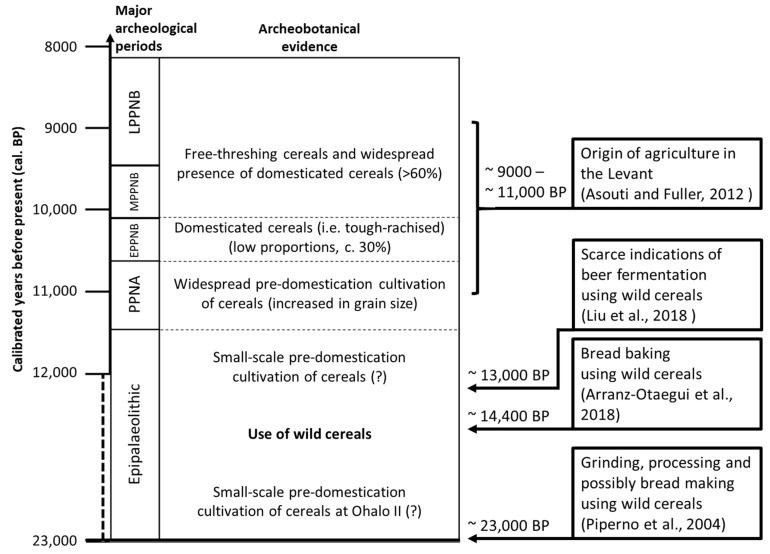
Cereal domestication and origin of agriculture in the Levant, with selected examples of exploitation of wild cereals dated to the time before the origin of agriculture. Major archaeological periods: Pre-Pottery Neolithic A (PPNA), Early, Middle and Late Pre-Pottery Neolithic B (EPPNB, MPPNB and LPPNB). See text for further details [4,14,18,20].

Finally, starch granules from bedrock stone mortars at the Raqefet Cave in Israel dated to 13,000 years BP suggests that even fermenting beer from wild cereals may have been practiced, and that beer was consumed in relation to mortuary rituals at the site [20]. All these finds date to before the onset of the actual farming of domesticated cereals, which is observed in the middle-to-late Pre-Pottery Neolithic period in the Levant around 9000–11,000 years ago [14]. These utilizations of wild plant species in the Levant are summarized in Figure 2.

For potatoes, the emergence of domestication traits is more complex and less documented in archaeological records than those of small-grain cereals. However, the consensus is that domesticated potato species originated in the south-central Andean highlands. Genetic analysis suggests a monophyletic origin of domesticated *Solanum tuberosum* [21]. The earliest record of potato consumed in Europe is found in the written records of a hospital in Seville, Spain, dated to 1573. However, potato production and consumption in Europe did not grow to significant levels until the 18th and 19th century [22]. With the Irish Potato Famine, caused by late blight in the mid-19th century, and its severe impact on health and the economy, it became evident that there is a necessity for potato varieties with increased resistance to *Phytophthora infestans*, the pathogen that causes late blight. Additionally, since the beginning of the 20th century, potato breeding has been using wild germplasm to introgress R-genes into potato cultivars [23]. An early example of the use of wild germplasm was the discovery of a potato hybrid between *S. demissum* and *S. tuberosum* in the Edinburgh Botanical Garden in 1910. This hybrid showed a particularly high resistance to *P. infestans* [22]. Later, genetic mapping identified that eleven dominant R-genes from *S. demissum* had been introgressed into cultivated potato varieties by hybridization [24,25]. However, the effect of the introgression of resistance genes from wild germplasm in potato breeding for control of late blight is often short, as new races of *P. infestans* develop to overcome cultivar resistance acquired through the introgression of wild resistance genes by hybridization. In contrast, by stacking R-genes from wild *Solanum* species in cultivars by cisgenic or transgenic technology, durable resistance has been acquired [26]. However, due to negative political and consumer perception of plants regulated as genetically modified organisms (GMOs), it is unlikely that crops produced by such techniques will be commercially viable, if such resistant varieties continue to be regulated as GMOs. Despite that, cultivation of such varieties is likely to reduce the need for pesticide use. An alternative way to use wild potato species with resistance to new pathogens is to integrate them into farming by de novo domestication. The prerequisite for this is that domestication alleles in cultivated potatoes are known.

## 2. De Novo Domestication: An Emerging Trend in Plant Science

De novo domestication is the process of domesticating wild or marginal crops by modern breeding techniques. This is a much-accelerated process compared to the speed of prehistoric domestication [27,28]. The change in morphological traits that occurred during domestication in prehistory can sometimes be partly reconstructed from comparisons of archaeobotanical finds from separate places and times in prehistory [29]. However, not all domestication traits can be observed in archaeobotanical finds, either due to the lack of preservation of the plant organ that bears the trait, or if the trait is simply a physiological character, which can only be observed in living plants. The grains and fruits of domesticated plants are larger than the wild crop relatives from which they have evolved by selection under cultivation. A domestication trait, particularly prominent for cereals, is the development of non-brittle rachis, which has led to the loss of natural seed scattering. Non-brittle rachis and seed size are some of the traits that can be scored in many archaeobotanical records, and the consensus is that the development towards larger grains and non-brittle rachis was a protracted process that occurred over a time of several centuries or even millennia [29], despite the fact that the non-brittle trait is controlled only by a single locus in barley encoding the two genes *Brt1* and *Brt2*. A loss-of-function mutation in either of these genes results in the non-brittle trait. The cloning and identification of these brittle genes in barley has made it possible to introduce this trait in wild barley accessions by mutagenesis [30]. Screening of a mutagenized population of 10,000 M_2_ plants derived from a seed population of brittle *H. spontaneum* treated with 1 mM sodium azide was enough to identify several mutants with non-brittle rachis, all of which had loss-of-function mutations in either the *Brt1* or *Brt2* genes. Other tools for re-domestication besides chemical mutagenesis include genome editing and selection on phenotypes. Genome editing has the additional advantage that it directs mutagenesis directly to the gene(s) of interest, whereby thousands of random mutations targeting other genes in the genome are omitted. Using these techniques that directly target key domestication genes, the speed of de novo domestication of a wild species in modern times may be less than 10 years.

The Idea that species can be domesticated by targeted mutagenesis has led to the exploration of genetic roadmaps that identify potential target genes to obtain traits necessary or propitious for crop cultivation [31]. For wild barley (*H. spontaneum*), Hanak et al. [27] suggested targeting the domestication genes responsible for non-brittle rachis (*Brt1*, *Brt2*), free threshing (*thresh-1*), naked grains (*Nud1*), six-row spikes (*Vrs-1*), plant height (*Apetala1, Dep1*), grain size (*emp5*) and grain weight (*TGW6-3*). These traits are typical for domesticated cereal species. For potatoes, a typical tuberous crop, [32] suggested targeting self-compatibility (*SRNase*), tuberization under long days and compact plants (*SP5G, CDF1*), low glycoalkaloid content (*GAME*—glycoalkaloid metabolism genes) and short stolons (*GA20ox1*). We note that whereas there are several review papers that discuss opportunities to re-domesticate or de novo domesticate wild, feral or marginally domesticated crops with new breeding technologies, only a few works report to actually have performed the task. Giving a full overview of wild or feral species previously suggested as candidates for domestication, as well as an overview of plants that have actually been de novo or re-domesticated by genome editing is beyond the scope of this paper, and can be found in several previous publications [27,33,34,35,36].

## 3. Another Brick in the Wall: The Knowledge Gap of Semi-Domesticated and Neglected Species

The Pleistocene (~1.8 million years BP–11,000 years BP) is characterized by the emergence of various hominid species including modern *Homo sapiens*. From the Late Pleistocene and into the Holocene, paleontological records witness a rapid extinction of large terrestrial vertebrates. This is coincident with the Epipaleolithic era, where human subsistence dispersed from an origin in the African continent to cover all the globe’s continents (except for Antarctica). While climatic changes may have been a component of the Late Pleistocene mass extinction of megafauna, high-resolution modelling of the timing of human arrival to world regions suggests that the anthropogenic impact through the hunting and killing of big game by Paleolithic *Homo sapiens* was the major cause of this mass extinction [37]. As the herds of big game disappeared, the late Epipaleolithic was characterized by an increase in the number of wild plant and animal species exploited by human hunter–gatherers, which eventually led to a few of those species being domesticated [38]. It has been suggested that this synchronicity between the extermination of large terrestrial animals due to human hunting and the origin of cultivation of small-grain annuals is linked to a shift in seed dispersal [39]. While several of the wild ancestors of domesticated annual plants had endozoochoric seed dispersal, the mass extinction of large vertebrate herds opened a mutualistic interaction between humans and dense accumulations of herbaceous annuals. However, rather than endozoochoric seed dispersal, the interaction with humans eventually shifted from gathering, transportation and food preparation of seeds to include deliberate cultivation, without endozoochoric seed dispersal at any stage. Other theories elucidate complex social causalities leading to the origins of plant domestication. Hayden [40] suggested that sedentary complex hunter–gatherers with surplus food productions may have practiced rites of competitive feasting leading to a demand for highly prestigious food and drink products, which may have included the production of alcohol from wild cereal grains, which eventually prompted grain production by cultivation. This is backed by the observations that larger efforts were put into the process of bread baking and indicates that beer production may have been practiced in the late Natufian, a hunter–gatherer culture which was present in the Middle East in the period 13,500–11,500 BP, just prior to the emergence of the first farming Neolithic cultures [18,20] (Figure 2). It has also been suggested that the invention of baking in prehistory has been a means to increase glycemic index (a measure of how much specific foods increase blood sugar levels) and, therefore, the amount of dietary energy available from grains, compared with that of raw or simply cooked grains [18]. Whether motivated by social or physiological means, a desire for an increase in dietary glycemic index most likely influenced the selection and evolution of crop grains, either as they were collected from wild stands or selected under cultivation. This is supported by the observation that breads baked from flour of *H. spontaneum* grains have a glycemic index that is more than 85% lower than that of breads baked from the flour of domesticated *H. vulgare* grains [41]. The lower glycemic index of *H. spontaneum* observed in this study was an effect of a complex inhibition of starch degradation that was neither caused by protein inhibitors nor low-molecular-weight inhibitors of starch hydrolytic enzymes, but rather a mechanism of higher dietary fiber content causing a decline in the viscosity of chyme.

**Figure 3:**
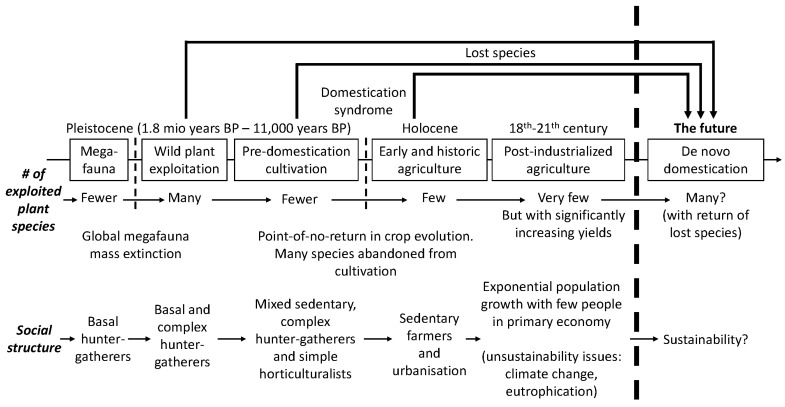
Human exploitation of plant species from the Pleistocene to modern times. Whereas the number of plant species exploited by humans may have increased as a repercussion of the global megafauna extinction towards the end of the Pleistocene, the general inclination in the pattern of human plant utilization was a significant decline in the number of plant species from many to very few species. The major event that led to a reduction in species exploited by humans was the onset of plant cultivation, where many foraged species were abandoned and only a few species evolved the domestication syndrome and reached major levels of cultivation. At the time of exponential growth following industrialization, humanity was dependent on the few species determined by domestication in the early Neolithic agricultural centers. The Green Revolution in the 20th century did lead to an increase in the yield of these major crops but did not see a notable change in the number of crop species. On the contrary, the transition towards modern intensive agriculture has increased the risk of varietal loss of the high agro-diversity known in traditional agriculture. With de novo domestication, it is now possible to significantly increase the number of crop species in agriculture by bringing in wild species that were abandoned in the deep past. Therefore, the future may have the advantage of agricultural production with a much higher plant diversity than we know now.

Wild grain exploitation in the Levant changed at the very end of Pleistocene and at the beginning of Holocene, where the first events of pre-domestication cultivation took place in the prehistoric era known as the Pre-Pottery Neolithic, dating from c. 12,000–c. 8500 BP. A systematic review on archaeobotanical finds throughout the Levant suggests that before systematic cultivation, many species of large-to-medium-sized wild grasses were gathered (and marginally cultivated?). This includes finds of grains from the genera *Aegilops*, *Avena*, *Bromus*, *Echinaria*, *Eremopyrum*, *Hordeum* (other than *spontaneum*), *Lolium, Piptatherum, Secale* and *Stipa*. However, with the emergence of widespread cultivation in the PPN era, wild relatives of diploid wheats (*Triticum boeticum/monococcum*/*urartu*) and barley *(H. spontaneum*) became dominant. This demonstrates a general decline in the number of species exploited by humans (Figure 3). It is an enigma why only a few of the *Poaceae* species became dominant among the annual wild species, and why only a few of them eventually were domesticated. In the transition from gathering wild grains from wild stands to the cultivation of domesticated species, several barriers must be broken down. A chain of operations needs to be carried out for cultivation to work. It must be possible to collect/harvest seeds or other botanic material that can be propagated, such as tubers. Dormancy must be timely, so that the material stays dormant—yet preferentially free of pests and microbial pathogens—until sowing, where a high germination frequency is preferred. The plant (or at least one organ containing dietary starch, lipid and/or protein) must not contain too-high concentrations of toxic compounds.

All archaeobotanical analysis suggests that the cultivation of plants for food originated among species that were gathered for nutritional purposes prior to their domestication. Naturally, some of those species possessed some of the traits mentioned above, whereas others appear to have evolved from a selection of domestication alleles under cultivation. It cannot be ruled out that those few species that became dominant in prehistorical agriculture and remain dominant today simply were domesticated because mutations leading to favorable domestication traits developed at random in those species and not in other species. In other cases, physiological traits in wild populations may have been a barrier to domestication. Perennial grass species have been systematically deselected over annual species. It is possible that annual species evolved quicker due to a higher number of generations in the same span of time, but it cannot be ruled out that the decline in megafauna and transition to seed dispersal by humans favored annual species before the onset of cultivation.

There are numerous examples of parallel domestication events of wild or closely related species. It is still debated if barley was domesticated only in the Levant or if there are multiple geographic origins [42]. Archaeobotanical records reveal many species that may have been cultivated in prehistory, which were later abandoned or deselected for cultivation (Table 1). Some of those also reveal examples of parallel domestication events of similar species between the world’s independent centers of agricultural origins. This speaks in favor of some wild species having a particularly high potential for domestication, either due to existing traits in the wild or, theoretically, also due to a certain plasticity in their genome, allowing them to develop domestication traits. For example, the exploitation of wild *Hordeum* species (other than *H. spontaneum*) is known from numerous finds in centers of agricultural origins in Eurasia and northern Africa. However, wild indigenous *Hordeum* species were also utilized in prehistoric North America. Eventually, this utilization led to the domestication of the species, little barley (*Hordeum pusillum* Nutt.), which is found in an indigenous crop complex in eastern North America dating to 3800 BP. that also included domesticated species such as bottle gourd (*Lagenaria siceraria*), sunflower (*Helianthus annuus*) and chenopod (*Chenopodium berlandieri*) [43]. However, little barley (*H. pusillum* Nutt.) was later neglected in agriculture, since there are no records of this species having been cultivated in more recent history. Similarly, there are signs of cultivation of the wild relatives of the domesticated potato (*S. tuberosum*) independent of its domestication in the Andean region in South America. Domesticated plants from Mesoamerica, including maize, entered the U.S. Southwest between 4000 BP and 1500 BP [44,45]. Additionally, it has been suggested that *Solanum jamesii*, a wild relative of *S. tuberosum,* was among them. Finds from archaeological sites in the Four Corners region of the U.S. Southwest—the states of Arizona, Colorado, New Mexico and Utah—suggest that Ancestral Puebloans in this region cultivated and processed foods from *S. jamesii* [44]. However, signs of use of *S. jamesii* tubers are much older than that. Finds of starch granules from tools used for grinding and food processing dated to 10,900–10,100 years BP. indicate tuber use in the area of southern Utah. Some of these starch granules can be assigned to the exploitation of wild tubers of *S. jamesii* [46]. *S. jamesii* is also known as the Four Corners potato and is marginally cultivated in present times. However, it may have been neglected for longer periods in the time frame from its earliest use in prehistoric times up to the present time. Another example of the parallel cultivation and domestication of closely related species in independent geographic regions in prehistory can be identified within the *Chenopod* family. Quinoa (*Chenopodium quinoa*), originally domesticated in South America, is a crop that has been met with increased interest from consumers and agricultural scientists in the last two decades due to its genetic variability and nutritional potential [47]. However, there are several examples of parallel domestication events of other wild *Chenopodium* relatives. In addition to the prehistoric cultivation of *Chenopodium berlandieri* in North America mentioned above, *Chenopodium album* is widespread in archaeological finds of Scandinavian prehistory. It is unclear if these finds represent intentional cultivation or if *C. album* was regarded as an undesirable weed, like it is in present-day agriculture. Finds of large stores of purified *C. album* seeds [48] from the early Scandinavian Iron Age (500 B.C.–A.D. 200) indicate that this species could have been cultivated deliberately, or was collected for nutritional purposes as a byproduct in connection with the threshing and cleaning of cereal crops [49]. The intake of *C. album* seeds in the Scandinavian Iron Age is supported by the find that Danish bog bodies from the early Scandinavian Iron Age such as the Grauballe man and the Tollund man carried such seeds in their stomach contents. However, the *C. album* seeds in the stomach contents were found together with other weed species seeds, charcoal and sand, indicating that they represent threshing waste rather than deliberately cultivated plants. The weed seeds could be a part of the staple food, but it is more likely that they represent a rare occasion, where the seeds were consumed for ritual purposes in connection with human sacrifice [50]. In summary, the prehistoric transitions from hunter–gatherer cultures to farming Neolithic cultures show a global pattern of a funneling from the use of numerous wild plant species to a gradual decline in the number of plant species exploited by humans. During the early formation of Neolithic farming cultures and in the events of the pre-domestication cultivation of plant species, numerous species may have been marginally cultivated. However, there is a gap in our knowledge of these crops, since only a few of those species survived through time, and the majority were neglected or abandoned, leading to independent farming cultures that were dependent on a limited number of species of stable crops. This pattern has remained until the present day. A non-comprehensive list of examples of plant species, where archaeobotanical studies indicate human exploitation in prehistory, but knowledge regarding cultivation or consumption has been lost, is given in Table 1.

## 4. Breaking down the Wall: From Plant Breeding in the 20th Century into a New Era of Agriculture with De Novo Domestication and Accelerated Domestication by Genome Editing

**Figure 4:**
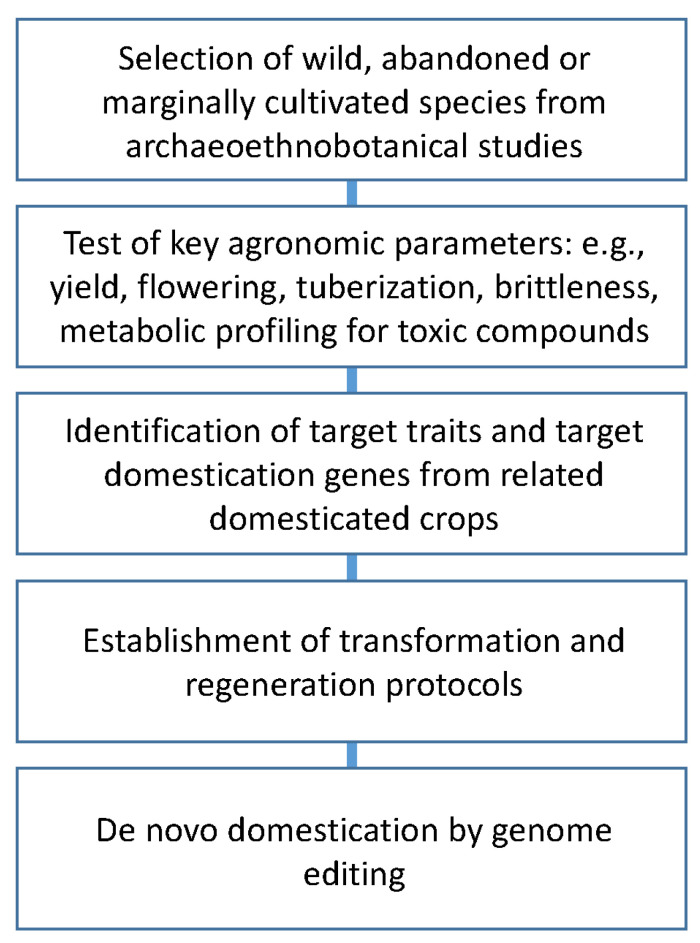
Generalized roadmap for selection of crops for de novo domestication.

With the emergence of modern science, new breeding tools ensued. The concepts of genes, genotype–phenotype correlation and gene linkage appeared with the breakthrough of Mendelian genetics. Chemical and physical mutagenesis then paved the way for the development of new alleles, of which many have been essential for traits in modern crop varieties. The term ‘The Green Revolution’ refers to the agricultural development in the second half of the 20th century—in particular from 1960 to 2000—where agricultural productions doubled [53,54] concurrent with an increase in the human population from around 3 billion people to more than 6 billion people [55]. This increase in yields during the Green Revolution has been estimated to have saved millions of hectares of natural ecosystems from being converted to agriculture. Under a scenario of omission of these increases in yield from breeding that the Green Revolution brought around, it is further estimated that the negative impacts of higher food prices on poverty and hunger would have diminished the welfare effects of agricultural expansion [55]. A pioneer in crop breeding, Norman Borlaug was awarded the Nobel Peace Prize in 1970 for his contribution to the Green Revolution through his efforts of breeding towards higher yields, or, in the words of The Nobel Foundation: “for having given a well-founded hope—the Green Revolution”. The genes that were targeted in Borlaug’s breeding program were primarily responsible for pathogen resistance and short plant stature. Changes in the latter trait were so significant that differences in the height of wheat plants can be seen in historic depictions of fields [56]. In his own words, Borlaug regarded his efforts as “a temporary success in man’s war against hunger and deprivation” (Norman Borlaug—Biographical—NobelPrize.org [57]). Indeed, breeding with random mutagenesis and crossings with existing germplasm, as well as the introduction of new genetic approaches such as hybrid cultivars, have continued to contribute to an increase in yields beyond Borlaug’s pioneering work [54]. However, the breeding potential within the few major crops upon which humanity relies is slowly dissipating, calling for novel solutions to generate new crop diversity. In this context, our times offer a unique opportunity to introduce new plant species into agriculture by de novo domestication. In line with this, and as shown above, the past saw a much higher number of plants exploited by humans. The study of these plants may assist in reintroducing them into the human diet. The combined effort of archaeoethnobotanical studies and modern plant breeding offers us the opportunity to pursue this task (Figure 4).

The archaeobotanical identification of wild plant species utilized by humans in the past can be complemented by the collection of extant accessions in the wild for agronomic testing to decide what genes to target for de novo domestication. Just as the Neolithic revolution introduced a new ecological basis for human existence, agriculture, the re-introduction and de novo domestication of lost and neglected crops from the past and from other wild species can herald a new revolution in human ecology with a significantly higher number of cultivated plant species and higher biological diversity. However, this is dependent on modern breeding being able to overcome barriers in the wild plants, such as anti-nutritional factors and low yields, to be able to compete with present-day high-yielding varieties.

In addition to this, it should be emphasized that there are many tropical crops still undergoing the process of domestication, which indicates the potential for further improvement of yield by breeding. Sarkar et al. [58] list 26 tropical fruits and describe their nutritional composition and potential as a direct food source of high diversity. In addition to crops that have been domesticated in (pre)history but later abandoned and lost, it has been suggested that there are a number of wild feral plants, crops which have escaped from cultivation [33]. Such plants are often regarded as geobotanical indicators of the historic or prehistoric human disturbance of a habitat. However, such plants also possess the potential for re-domestication. Millets are a complex group of several species belonging to the Poaceae family, some of which exist both as cultivated species and in wild stands. They are among some of the earliest domesticated crops, and archaeobotanical analysis [59] has shown that common (also known as broomcorn or proso) millet (*Panicum miliaceum*) and foxtail millet (*Setaria italica*) were domesticated in Neolithic China dating as far back as between 10,300 and 8700 years BP for *P. miliaceum*. A meta-study of archaeobotanical finds mapping prehistoric cereal migration in Eurasia [8] supports this conclusion, suggesting that the East Asian millets *P. miliaceum* and *S. italica* arose and spread from a region in present-day China, whereas African millets arose in at least two independent centers in the northernmost part of sub-Saharan Africa. By 2500 B.C., both East Asian and African millets had spread over a larger area of Eurasia. In 2005, Pami Kothari et al. [60] discussed the many potentials of 14 species of millet in a review assessing the status of biotechnological methods accessible to improve them. However, by 2022, a similar review discussing genome editing in millets concluded that the new breeding technologies for these species are still limited by the lack of investment in efficient transformation systems and the lack of bioinformatic studies on these species [61]. Before genome editing and new breeding technologies existed, it was attempted to domesticate wild germplasm by other means. Crop hybridization with wild species is an example of how studying the effects of prehistoric crop domestication has inspired a science-based creation of new crops. One of the world’s most common cereals, bread wheat *(Triticum aestivum),* having a hexaploid genome AABBDD, is known to be the result of a prehistoric spontaneous hybridization event between tetraploid *Triticum turgidum* donating the A and B genome, and *Aegilops tauschii* donating the D genome [62]. Tritordeum is a hybrid between wheat and the wild barley *Hordeum chilense,* developed by plant breeders in the 20th century, and is now produced in its hexaploid version by farmers around the world. It has significant qualities of resilience towards biotic and abiotic stresses, originating from the wild barley genome; additionally, it is of acceptable baking quality [63]. The success of this crop demonstrates how modern science-based plant breeding can be used to domesticate resilient wild germplasm for the benefit of introducing novel crop species [64]. Increasing the number of crop species that are available for cultivation offers an opportunity to meet several novel goals for plant production. Intercropping is an agricultural practice where multiple crops are grown together in the same field. As reviewed by Maitra et al. [65], intercropping is not a new idea, but has already been practiced earlier in history. It has the potential to provide several benefits, including improved soil health, improved carbon storage in the soil and more efficient use of light, water and soil mineral nutrients. It may also be a means to reduce pest and disease pressure, and generally increase overall farm productivity, with less need for input such as mineral nutrients. Existing crop species are usually produced in monoculture and may not be optimal for intercropping. Plants that have been exploited by humans in prehistory but that were never domesticated, as well as feral species and minor crops, may have good potential to be produced in intercropping systems by introducing the necessary traits using genome editing for accelerated domestication or for de novo domestication. Increasing the number of crop species for food may also support the variety of raw material for plant-based foods and vegan diets.

## Figures and Tables

**Table 1:** List of examples of lost species that were exploited in prehistory but were never (fully) domesticated.

Species	Location and Date	Usage	Reference
*Hordeum pusillum* Nutt.	Riverton Site, Illinois, USA, 3800 BP	Indication of cultivation	[43]
*Chenopodium berlandieri*	Riverton Site, Illinois, USA, 3800 BP	Indication of cultivation	[43]
*Solanum jamesii*	Four Corners region, USA, 4000 BP–1500 BP	Indication of cultivation	[44]
*Chenopodium album*	Denmark and northern Germany, 500 B.C.–A.D. 400	Systematic gathering or possible auto-cultivation. Dietary usage, possibly for ritual purposes	[48,49,50,51,52]
*Persicaria maculosa*/*lapathifolia sl.*	Denmark and northern Germany, 500 B.C.–A.D. 400	Systematic gathering or possible auto-cultivation. Dietary usage, possibly for ritual purposes	[49,50,51,52]
*Fallopia convolvulus*	Denmark and northern Germany, 500 B.C.–A.D. 400	Systematic gathering or possible auto-cultivation. Dietary usage, possibly for ritual purposes	[48,50,51]
*Spergula arvensis*	Denmark and northern Germany, 500 B.C.–A.D. 400	Systematic gathering or possible auto-cultivation. Dietary usage, possibly for ritual purposes	[49,50,51,52]
*Bromus secalinus*	Denmark and northern Germany, 500 B.C.–A.D. 400	Possible auto-cultivation	[51]

## Data Availability

No new data were created or analyzed in this study.
